# Editorial Correspondence

**Published:** 1871-11

**Authors:** W. F. Westmoreland

**Affiliations:** New York


### EDITORIAL CORRESPONDENCE.

NEW YORK, October 5th, 1871.

*Dear Journal:*—In a previous letter I mentioned, if I am not mistaken, Dr. Sayre's absence in Europe. He returned a few days ago, much gratified at his reception by the profession in the great cities of Europe. I very much regret that time and space will not permit me to give the many gratifying incidents of his trip. In England, Scotland, Ireland, France, Prussia, France, and in fact wherever he went, the compliments tendered him were certainly sufficient to satisfy his most enthusiastic friends, and to make every medical man of this country feel proud of their representative and the honors conferred on him.

In London, he was solicited by the Governors of the prominent Hospitals to lecture on the treatment of hip-joint disease, an invitation rarely extended to the most distinguished medical men. His lectures were reported in full in the London journals, with compliments exceedingly flattering to the author.

Thirteen or fourteen years ago, when he was less known to fame, and when he was struggling to establish the same great principles which now make him so justly eminent, in a letter from this city to the ATLANTA MEDICAL AND SURGICAL JOURNAL, I predicted his brilliant future. It is exceedingly gratifying, to-day, to see my predictions more than realized. I was sorry to find him confined to his room from a sprained ankle, the result of an injury received in Ireland just before sailing. In every other particular, he is in fine trim—is now in the prime of life, and as enthusiastic in all that pertains to his profession as he was fourteen years ago.

A favorite resort with me, on former visits to New York, was the Woman's Hospital; but during my entire stay this time, it has been closed for repairs. I have had, however, the pleasure of meeting its distinguished Surgeon-in-Chief, Dr. Thomas Addis Emmett, on several occasions. The Doctor is the same bluff, candid man as heretofore. With a fine intellect, well stored with information, he is ever ready to give his opinions upon subjects connected with his specialty, and with a candor truly refreshing.

By request, I attended to-day the Surgical Clinic of Prof. Frank H. Hamilton, at Bellevue Hospital Medical College, to witness the treatment of two cases of cancer by electrolysis.

The first case presented by Prof. Hamilton was a woman between thirty and forty years of age, with cancer of the right mammary gland. The disease had existed for a year or more, and was unmistakably malignant. Ten days ago, Dr. A. D. Rockwell gave her the first treatment with the galvanic current, with results truly gratifying. Prof. Hamilton stated that the tumor had been reduced within that time fully one-half, and that the pain, of which she complained so greatly before the treatment, had entirely ceased. A small ulcerated point near the nipple had almost entirely filled up with healthy granulations. Dr. Rockwell, who was present, made another application of the galvanic current. As the subject is new, it would, perhaps, not be uninteresting to many to know some of the details of the application.

The woman was put fully under the influence of ether, and three needles, from three to four inches long, with platina points, and insulated with hard rubber to within a fourth of an inch of the point, were introduced into the cancerous breast at different points. The needles were then connected with the negative pole of a galvanic battery with twenty elements (Smee's elements), and the circuit made by means of a sponge electrode from the positive pole applied to the skin near the centre of the diseased gland. The application was commenced with only ten elements, which was gradually increased to twenty, the entire power of the battery. The current was continued about twenty minutes.

The other case was a woman between forty and fifty years of age, who for several years had suffered from a cancer commencing in the right breast. She had submitted to several operations, but after each the disease reappeared. She had now a hard tumor in the cicatrix of the last operation. The galvanic current was applied to-day for the first time, as in the case above mentioned.

Dr. Hamilton is not yet satisfied what can be accomplished in malignant diseases by electrolysis, but thinks the case just mentioned an undeniable evidence of beneficial results. In the first case presented, from one application, the tumor was reduced, in ten days, one-half, with immediate relief from all suffering. This, with similar results familiar to many in the city, is sufficiently encouraging to test its action further. He is not disposed to believe in the complete eradication of malignant disease by this process. The only case reported (that of Dr. Neftel's) where a sufficient length of time had elapsed to warrant this view, the disease, unfortunately, was wanting in some essential parts of its history to make an indisputable case of cancer.

Surgical electricity is exciting considerable interest here. Many of the prominent medical men of the city are testing its virtues; so we may expect, before a great while, to know something more definite on the subject. To-day I supplied myself with all the necessary appliances, and propose, upon my return to Atlanta, to investigate for myself.

Prof. Hamilton informed me that the Board of Physicians and Surgeons of Bellevue Hospital had, to-day, rejected a proposition from Dr. Bliss to furnish the physicians and surgeons in attendance, Cundurango for their patients suffering with cancer. It was rejected, I suppose, upon the idea that Dr. Bliss would use the acceptance of the proposition as an advertisement for the sale of the remedy. I have seen no one here who has any confidence in the remarkable and specific effects of the Cundurango in cancerous affections, as claimed by Dr. Bliss.

I have several times met Dr. A. D. Rockwell, and find him a quiet, intelligent and energetic young man, with more candor than is usually found in medical men who have been devoted for a considerable time to a specialty. He has, for the past several years, given his entire time to electro-therapeutics, and is one of the authorities upon that subject in New York. In connection with Dr. Beard, he has recently published an excellent work of six hundred pages on "The Medical and Surgical Uses of Electricity."

To-day I met, for the first time, Dr. C. R. Agnew, Clinical Professor of the Diseases of the Eye and Ear in the College of Physicians and Surgeons. He returned to the city two days ago, after an absence of several weeks. From very flattering reports from various sources, I was favorably impressed before I met him; the interview certainly greatly strengthened the impressions previously formed. Dr. Agnew stands at the head of the profession as an oculist, and is in every way reliable. I very much regret that circumstances prevented my seeing more of him, but on some future visit, hope to see some of his brilliant operations on the eye.

Through the courtesy of my friend, Dr. Gouley, a few days ago, I had the pleasure of meeting Dr. Meredith Clymer, late Professor of the Practice of Medicine in the University of New York. Dr. Clymer is a learned man, a fine talker, and has the reputation, in medical circles here, of writing with more facility and polish, than any medical man in the city. He is devoting the most of his time to the diseases of the nervous system, in connection with electro-therapeutics, and will, before a great while, give to the profession the result of his thorough investigation and long experience.

I very much regret that circumstances have prevented me seeing more of Dr. St. John Roosa, Clinical Professor of the Diseases of the Eye and Ear in the University of New York. Dr. Roosa is a hard worker, and enthusiastic in his profession. He is at present preparing a work on the "Diseases of the Ear," which is now passing through the press, and will appear sometime next year.

I shall leave for Georgia to-night, via Philadelphia, Cincinnati, Louisville and Nashville, and hope to be able to spend a day or two in each city. My stay here has been in every way pleasant. Never, in all my life, have I been treated with more kindness and consideration than by the profession of New York. My only regret is that duty compels me to leave.

Truly, W. F. WESTMORELAND.

